# Global Trade Tradeoff: Rickettsial Disease in Taiwan

**DOI:** 10.1289/ehp.120-a456

**Published:** 2012-12-03

**Authors:** Sharon Levy

**Affiliations:** Sharon Levy, based in Humboldt County, CA, has covered ecology, evolution, and environmental science since 1993. She is the author of *Once and Future Giants: What Ice Age Extinctions Tell Us about the Fate of Earth’s Largest Animals*.

After Taiwan joined the World Trade Organization (WTO) in 2001, local production of rice plummeted. A new study shows that fallow Taiwanese rice fields have become breeding grounds for ticks and chiggers that infect people with deadly diseases.^1^ However, plowing the fallow fields appeared to reduce the number of arthropod vectors carrying disease, relative to unplowed fields.

The WTO requires Taiwan to open trade in rice with less developed countries that produce the crop more cheaply. Imported rice therefore outcompetes local rice in the Taiwanese market, causing many farmers to abandon their rice fields. By one estimate, as of 2011 about 40% of the country’s rice fields were uncultivated.^2^ Remaining active rice paddies are small plots of 1 hectare or less, scattered among a patchwork of unused fields.

Ecologist Chi-Chien Kuo and colleagues from the University of California, Davis, and the Taiwan Centers for Disease Control set out to study the ecology of rickettsial diseases in the abandoned fields of Hualien County, once a major center of rice production. These diseases—including scrub typhus and spotted fever—take their name from the bacterial genus *Rickettsia*, one of the implicated pathogens. They are passed to humans through the bite of ticks, chiggers, and other parasites, and some can be fatal if left untreated.

Although Hualien is one of Taiwan’s least-populated counties, it has reported one of the country’s highest rates of scrub typhus, which is caused by the bacterium *Orientia tsutsugamushi* and transmitted by chiggers. “Since 2003 there has been a forty-percent increase in human cases of scrub typhus in Taiwan,” Kuo says. Spotted fever, caused by *Rickettsia* spp., is a new pathogen in the region that is transmitted by ticks.

**Figure:**
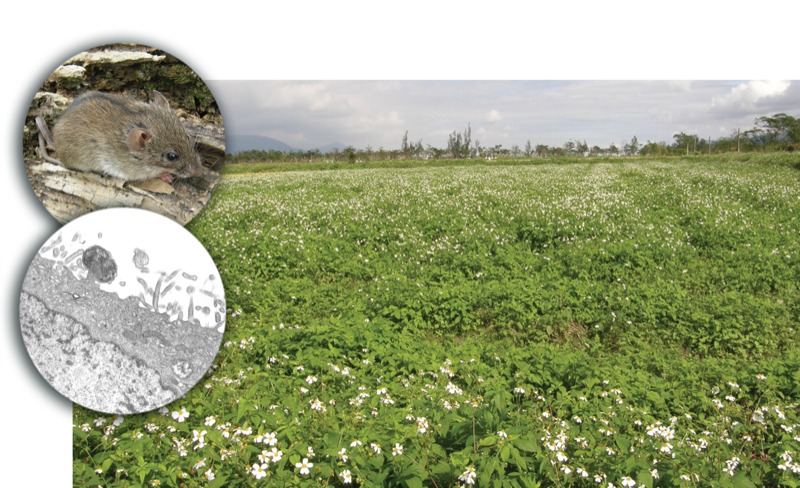
Abandoned rice paddies like this weed-filled field in Hualien offer prime breeding grounds for chiggers and ticks carrying pathogens (one such pathogen is O. tsutsugamushi, bottom inset). Striped field mice from unplowed fields carried many more chiggers and ticks than mice from plowed fields. Lower inset: CDC/Ed Ewing; upper inset and main image: Chi-Chien Kuo/University of California, Davis

The Taiwanese government subsidizes farmers who plow their unplanted fields, a strategy intended to control pests that could devour crops in neighboring rice paddies. Kuo and colleagues trapped rodents in plowed and unplowed unplanted fields, and collected ticks and chiggers by carefully combing through the animals’ fur. Because the arthropod vectors can’t survive under water, they are rare in cultivated rice paddies, which are flooded on a regular basis. But the team did not look at active rice paddies because of the difficulty of trapping rodents in flooded fields.

The researchers focused their analysis on the striped field mouse, *Apodemus agrarius*, which hosted the great majority of the ticks and chiggers they recovered. Mice from unplowed plots carried nearly three times more chiggers and over six times more ticks than mice from plowed fields. Moreover, the percentage of ticks that carried *Rickettsia* spp. was three times higher in mice from unplowed plots, although *O. tsutsugamushi* burden did not vary between groups.

Plowing exposes soil to the sun, drying it out. “Though both ticks and chiggers parasitize rodents, they spend most of their life cycle in the ground and are very susceptible to low soil humidity,” explains Kuo. His results show that while plowing had little impact on rodent numbers, it did have the unintended benefit of reducing populations of disease-carrying ticks and chiggers.

“This study is a great example of the kinds of indirect effects that trickle down from human policies,” says Bob Parmenter, director of the U.S. Department of Agriculture Scientific Services Division at Valles Caldera National Preserve, New Mexico. “It tells a nice story about how changes in international trade barriers can have unforeseen consequences.” Parmenter is an expert on the ecology of hantavirus outbreaks like this year’s event in Yosemite National Park.^3^

“This work makes a plausible case that land-use changes [in Hualien] following from joining the WTO increased the likelihood of human infection,” says Richard Ostfeld, a disease ecologist at the Cary Institute of Ecosystem Studies in Millbrook, New York. Ostfeld warns that changes in climate and human demography can also have a strong influence on infection rates, making it difficult to establish a cause-and-effect relationship between abandonment of rice fields and the increase in human cases of rickettsial disease. He also points out that the tick and chigger burden on rodent hosts provides only a crude proxy for the risk of human infection; at this life stage, arthropods that have already found rodents to bite are no longer searching for a mammalian host.

Plowing of unplanted rice fields seems to Kuo to be an expensive and unsustainable strategy. He believes cultivation of rice or other crops would be the best use of those abandoned fields in Hualien, given recent food shortages around the world, and would significantly reduce disease risk.

Taiwan’s rural population has been aging and shrinking for decades, as young people move to the cities, and under the terms of the WTO agreement, local rice farmers report not being able to make a decent profit. These and other factors have prompted the Taiwanese Council of Agriculture to frame new policies to turn Taiwan into a “Pesticide-Free Agricultural Island” by the middle of this century.^2^ For the near term, however, Taiwan has become dependent on imports for its supply of rice, a staple food, while disease-carrying pests multiply in the fallow fields. Kuo says, “Without a government subsidy or other economic incentives for rice growers, I can’t see any easy solution.”
