# Erratum: “Global Trade Tradeoff: Rickettsial Disease in Taiwan” [120(12):A456 (2012)]

**DOI:** 10.1289/ehp.121-a113a

**Published:** 2013-04-01

The photo caption in the December 2012 News article “Global Trade Tradeoff: Rickettsial Disease in Taiwan” (Environ Health Perspect 120:A456; doi: 10.1289/ehp.120-a456) incorrectly stated, “Striped field mice from plowed fields carried many more chiggers and ticks than mice from unplowed fields.” The caption should have read, “Striped field mice from unplowed fields carried many more chiggers and ticks than mice from plowed fields.”

*EHP* regrets the errors.

